# Native flowering plants and their pollinators for use in urban landscape: the Turkmen Mountain case

**DOI:** 10.7717/peerj.21143

**Published:** 2026-05-04

**Authors:** Hilal Topcu, Sibel Yigiter, Coskun Guclu

**Affiliations:** 1 The Parks and Gardens Department of the Eskisehir Metropolitan Municipality, Eskisehir, Odunpazarı, Eskisehir, Turkey; 2 Department of Horticulture, Agriculture Faculty, Osmangazi University, Eskisehir, Turkey; 3 Department of Agricultural Biotechnology, Faculty of Agriculture, Osmangazi University, Eskisehir, Turkey

**Keywords:** Native plants, Diversity, Convolvulaceae, Fabaceae, Climate

## Abstract

This article presents a comprehensive field study examining the interaction between native flowering plant species suitable for urban landscapes and the pollinators that visit them. The research was conducted in June 2021 at 13 different locations in the Turkmen Mountain region within the borders of Eskisehir. A total of 57 flowering plant species were identified in the study; pollinator activity was observed in 35 of them. Additionally, the species diversity of pollinators and their interactions with plants were examined in detail, and for this purpose, the Pollinator Activity Index (PAI) was developed, combining the diversity of plant families, pollinator visits, and pollinator diversity. The findings revealed that the Fabaceae family had the highest PAI value (2.484 × 10^−5^) compared to other plant families, and some other plant families, such as Convolvulaceae and Lamiaceae, also had significant interaction levels. Specifically, it was found that pollinator activity was optimal within a temperature range of 17–19 °C; humidity, wind, and solar radiation partially affected pollinator behavior. Furthermore, the high rates of morphological adaptation and phenological overlap of plants indicate that flowering periods and pollinator activity are well synchronized. In the discussion section of the article, it is emphasized that the conservation and preference for natural plant species in urban ecosystems are critically important for supporting pollinator populations, sustaining ecosystem services, and conserving biodiversity. It is also revealed that urban green spaces provide habitat and microclimatic refuges for pollinators; therefore, they play a decisive role in pollinator-plant interactions. In conclusion, the study provides a scientific basis for the development of plant selection and landscape design strategies in sustainable urban planning.

## Introduction

The plant–pollinator relationship is of critical importance for seed production and the sustainability of biodiversity in ecosystems. Approximately 75% of flowering plants depend on animal-mediated pollination, and nearly 35% of global plant-based food production relies on pollination services provided primarily by insects, particularly bees (Klein et al., 2007; Ollerton, Winfree & Tarrant, 2011; Ulus & Ozdemir, 2018). However, serious declines in pollinator populations have been reported in recent years (Le Buhn & Luna, 2021; Vasiliev & Greenwood, 2021; Barrett, Fischer & Buchmann, 2023). Bottero (2019) reported that between 1947 and 2005, honey bee colonies declined by 25% in Central Europe and by 59% in the United States. Modern agricultural practices, pesticide use, habitat fragmentation, climate change, reduced floral diversity, and the spread of diseases are considered the primary drivers of this decline (Kluser & Peduzzi, 2007; Guler, 2016). Understanding the ecological interactions that enhance ecosystem services is therefore essential (Garibaldi et al., 2018). In particular, accurate interpretation of plant–pollinator relationship is crucial for the restoration of degraded ecosystems and the recovery of ecosystem functions that support human well-being (Glenny, Runyon & Burkle, 2023). Ecological restoration offers opportunities to conserve biodiversity by ensuring the continuity of pollination services and promoting landscape designs that support sustainable agriculture (Bergamo et al., 2023).

Urbanization negatively affects pollinator communities through habitat fragmentation, increased impervious surfaces, pollution, and pesticide use (Fenoglio, Rossetti & Videla, 2020). For example, Theodorou et al. (2020) demonstrated that urban fragmentation reduces floral richness, which indirectly decreases bee species richness and abundance. Nevertheless, urban environments can unexpectedly support high levels of biodiversity through parks, gardens, and other green infrastructure elements. In some cases, cities have even been reported to host higher bee species richness than surrounding agricultural areas (Hall et al., 2017). However, caution is required when interpreting comparisons of pollinator richness between urban and natural or semi-natural systems. Recommendations for urban landscapes design based solely from data obtained from semi-natural or high-altitude ecosystems are inherently limited. Therefore, field-based empirical studies are needed to bridge the gap between urban development and ecological conservation (Dixon, 2009; Cariveau, Bruninga-Socolar & Pardee, 2020; Glenny, Runyon & Burkle, 2023).

The present study aims to identify native plant species with potential for use in urban landscapes, along with the pollinator groups associated with these plants, by examining flowering plant species and pollinator interactions in Turkmen Mountain, located within the borders of Eskisehir Province. In this way, the study seeks to contribute to the enhancement of urban biodiversity and the strengthening of pollination services in urban ecosystems. Climatic conditions in the study area play a dominant role in shaping plant–pollinator interactions. In June 2021, the average temperature in Turkmen Mountain was 17.8 °C, relative humidity was 71.9%, and total precipitation was 55.8 mm. These conditions strongly influenced both flowering phenology and pollinator activity. At the urban scale, the effects of climatic variables on these interactions must be carefully considered in landscape planning.

The theoretical framework and methodological contribution of this research is embodied in the development of a new mathematical indicator, the Pollinator Activity Index (PAI). PAI was designed to quantitatively evaluate pollinator activity by integrating biological and environmental components. In earlier conceptual formulation, placing environmental coefficients in the denominator could unintentionally penalize favorable environmental conditions. To address this issue, an alternative approach was adopted that rewards environmental quality by establishing a positive multiplicative relationship (*e.g.*, number of pollinators observed × environmental quality score). Under this framework, PAI attains higher values in structurally rich and environmentally suitable habitats, increasing its sensitivity to habitat quality.

The validity and ecological relevance of PAI should be evaluated through comparisons with established ecological diversity metrics. Previous studies have reported positive relationships between bee abundance, species richness, and floral richness (Plant et al., 2025; Sirohi, Jackson & Ollerton, 2025). Accordingly, the performance of PAI can be assessed by examining its relationship with species richness, Shannon diversity, and structural indices of plant–pollinator networks. To ensure transparency and reproducibility, all components of the PAI formula are explicitly defined, and detailed calculation procedures are provided in the Methods section.

Overall, this study investigates plant–pollinator interactions in Turkmen Mountain, a semi-natural ecosystem, while discussing the results as transferable baseline inferences for urban landscape design. The proposed PAI has the potential to quantitatively capture interaction dynamics between plant and pollinator communities, offering practical guidance for landscape architects, urban planners, and policymakers aiming to develop pollinator-friendly urban environments. In this way, the study contributes to building a knowledge-based bridge between urban development and ecological conservation.

## Study Area

**Figure 1:**
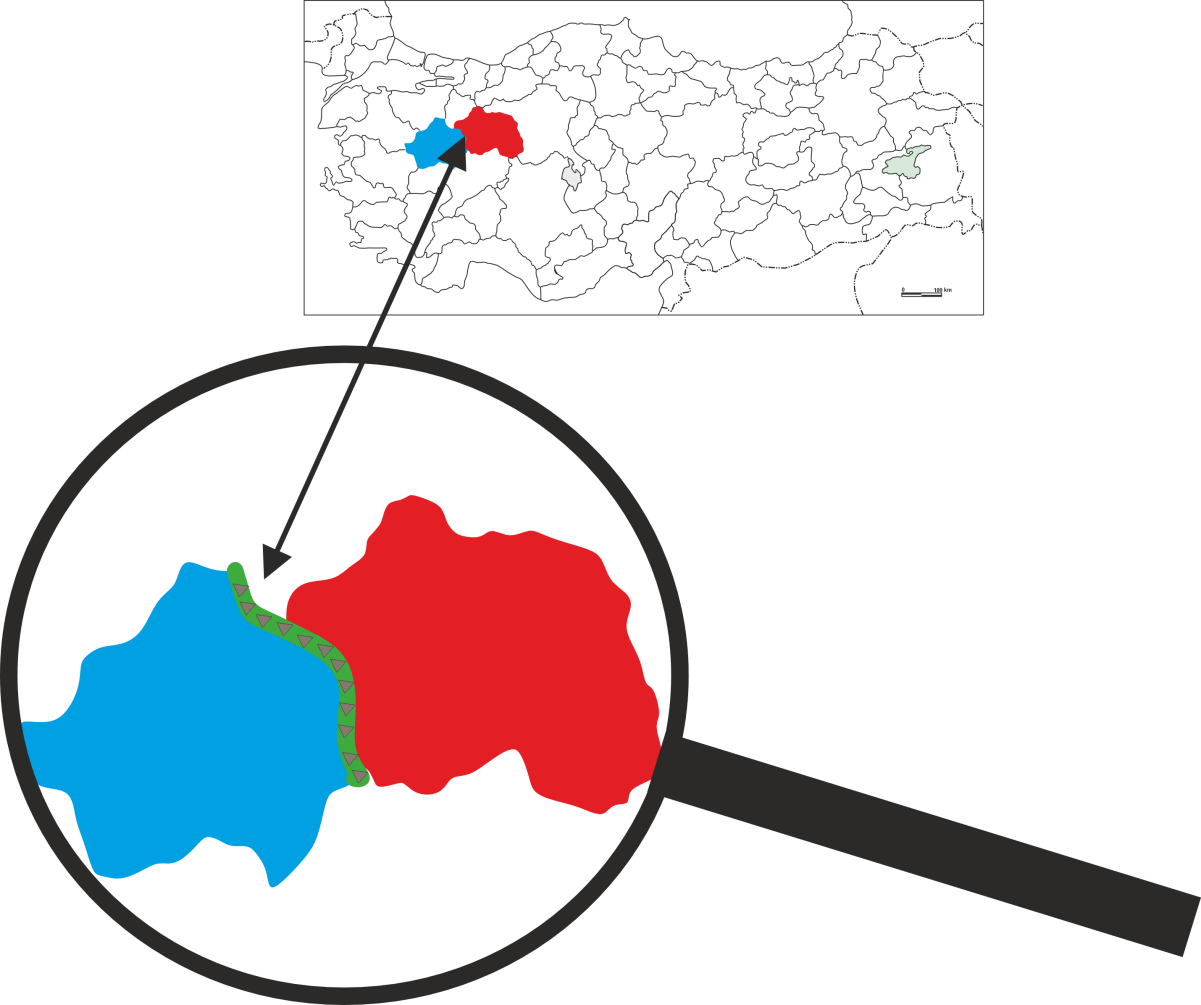
Location of the study area (designed by Coskun Guclu).

Turkmen Mountain is located between the provinces of Eskisehir and Kutahya (Fig. 1). To its west is Kutahya, to its east is the Seyitgazi district, to its south is Afyonkarahisar, and to its northeast is Eskisehir. The area, extending in a northwest-southeast direction, is surrounded by the Sundiken Mountains to the north, Sivrihisar Mountains to the east, Egrigoz Mountain to the west, Uludag to the northwest, and Emir Mountains to the southeast.

## Materials & Methods

**Figure 2:**
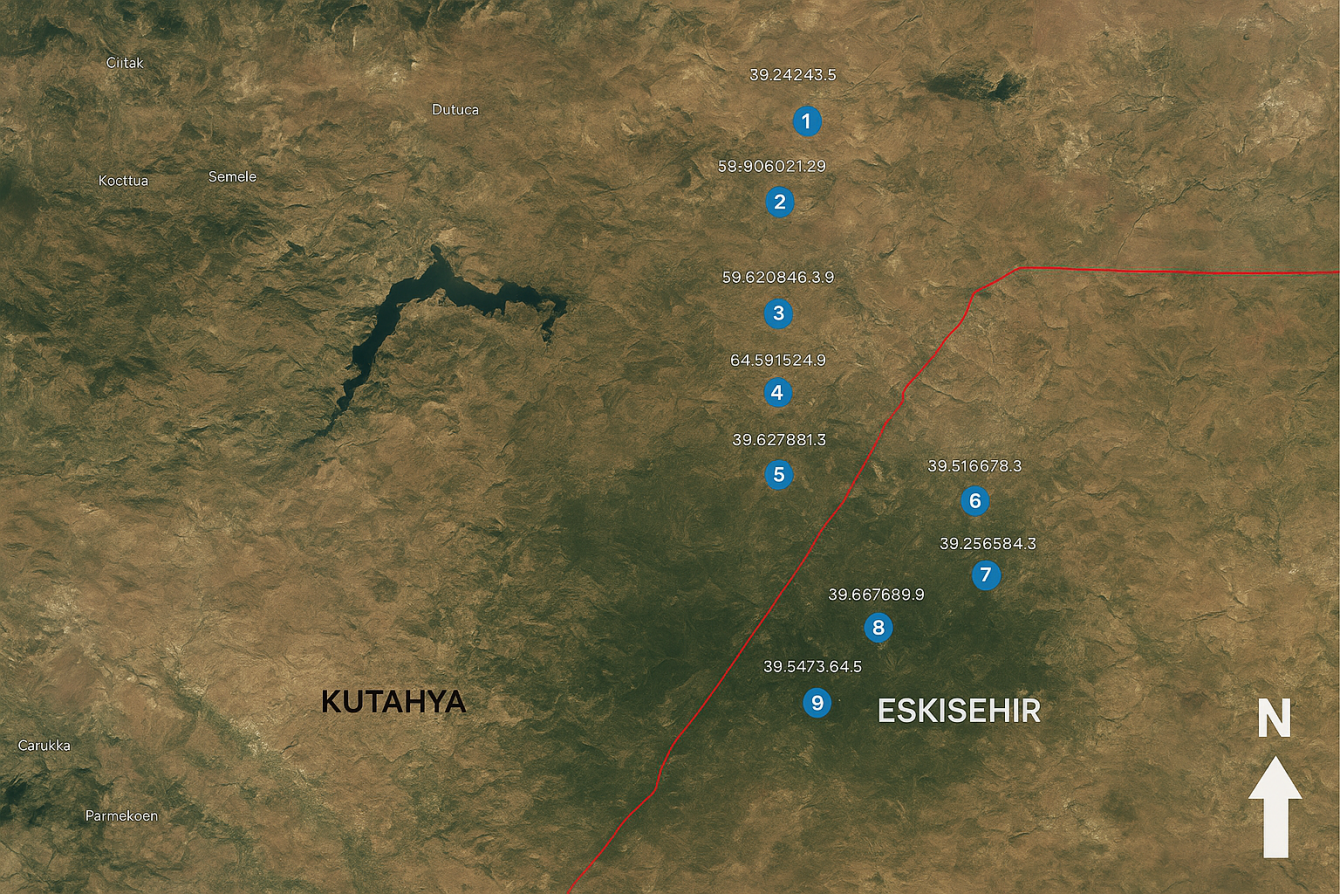
Observed locations (Google, 2026).

**Figure 3:**
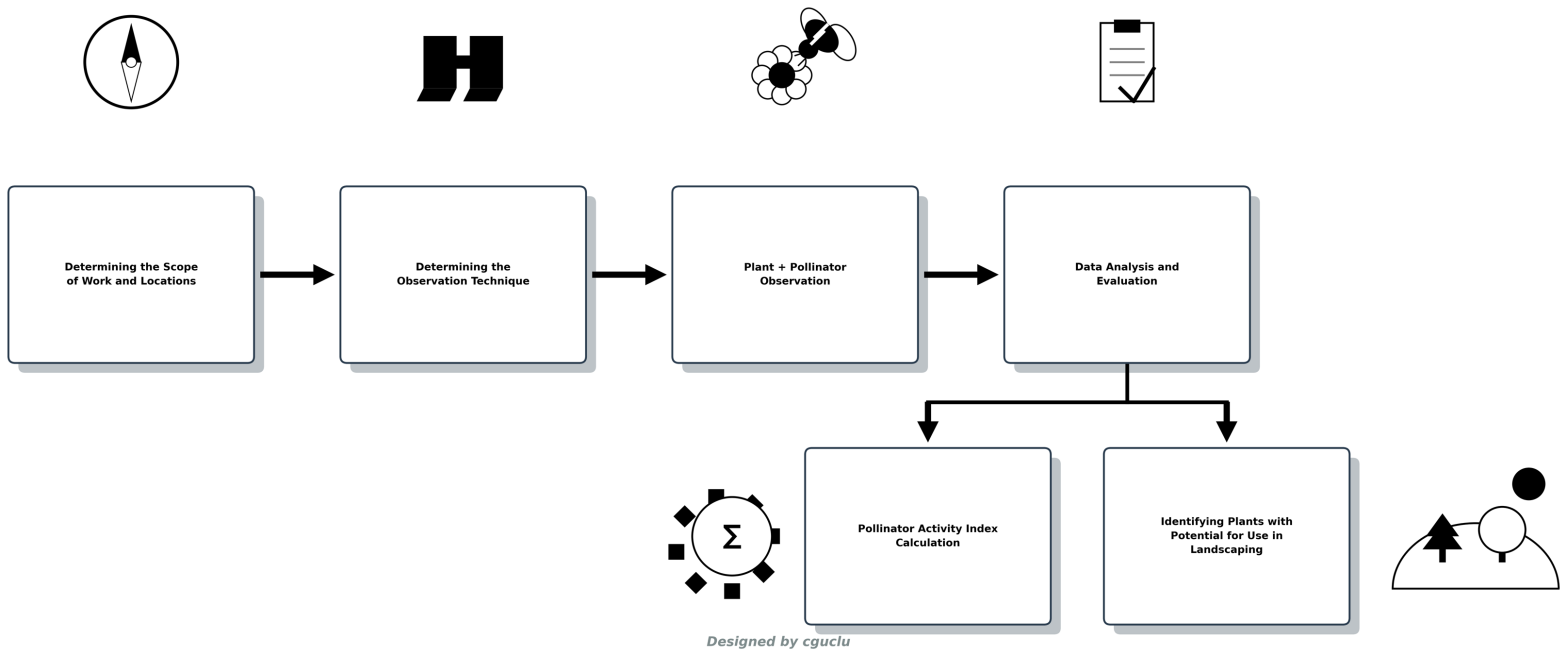
Method flowchart (designed by Coskun Guclu).

The research is based on systematic observations conducted at 13 different locations identified in the Turkmen Mountain region (Fig. 2). The observations were conducted in June 2021. This period was chosen because it coincides with the peak flowering season for plants, when pollinator activity is at its highest. At each location, the duration of pollinator visits to flowering plants, species diversity, and environmental factors (air temperature, relative humidity, total precipitation, and sunshine duration) were recorded in detail. Plant and pollinator species were identified using current botanical and entomological literature, and the potential use of each species in urban landscapes was evaluated. To quantitatively assess the pollinator attractiveness and activity of plant families, the Basic Pollinator Activity Index (PAI) was calculated and analyzed (Fig. 3).

### Study period and climate data

The field observations of the study were carried out in June 2021. Observations were conducted on sunny and rain-free days. During this period, the average temperature of the region was recorded as 17.8 °C, average relative humidity as 71.9%, total sunshine duration as 210.1 h, and precipitation as 55.8 mm. The soil temperature (at 20 cm depth) was measured as an average of 20.3 °C (Table 1). The study also examined the relationship between environmental parameters, such as temperature, humidity, wind speed, and solar radiation, and pollinator activity.

### Data collection and observation technique

**Figure 4:**
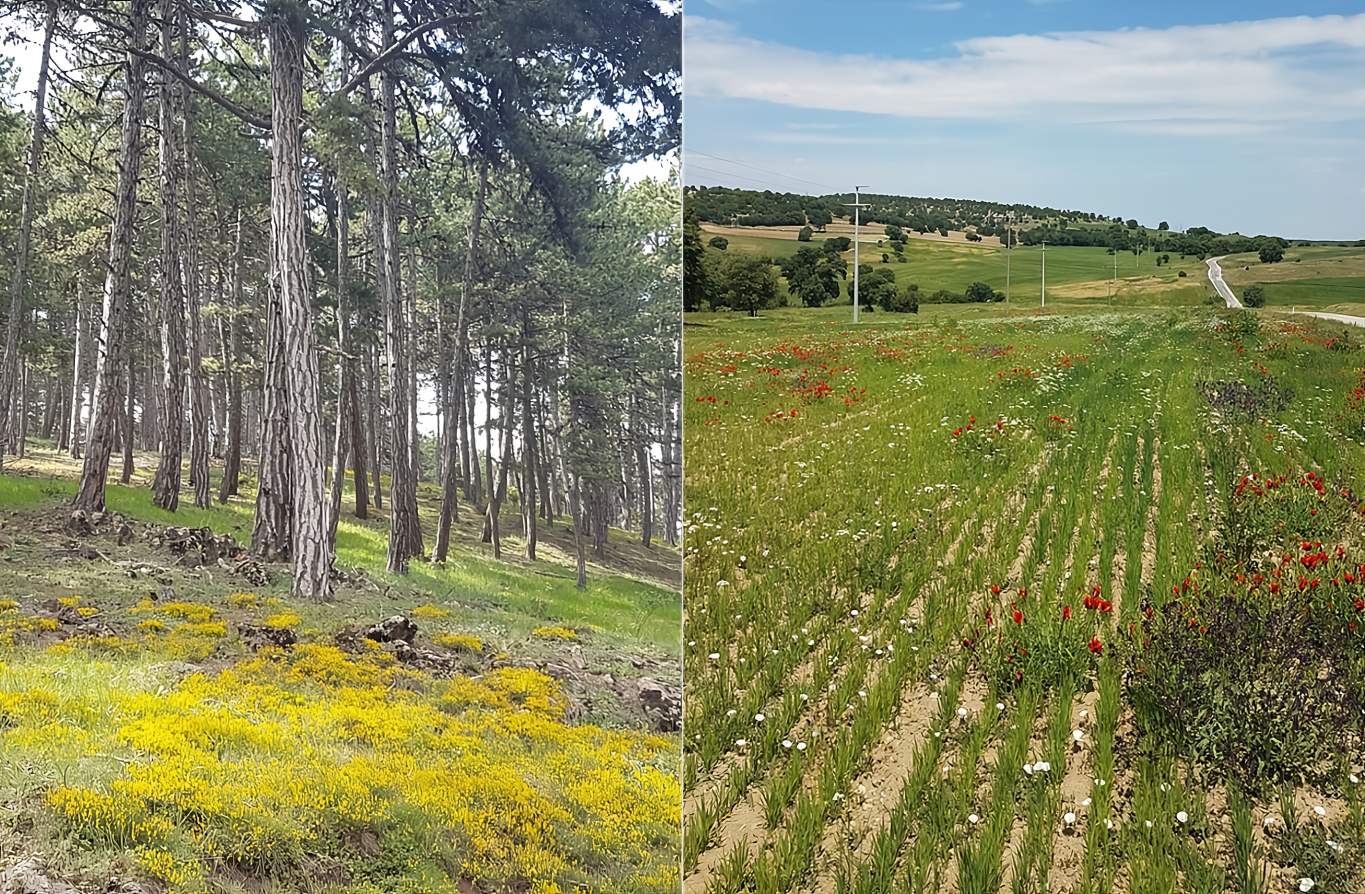
Representative habitats of the Turkmen Mountain study area where plant–pollinator interactions were recorded: (A) forest understory; (B) open and semi-open agricultural and roadside habitats (Orij, 2021).

Thirteen locations were identified in the study area to represent different habitat types (open areas, roadsides, agricultural areas, forest edges, *etc*.) that reflect the ecological diversity of the region and provide easy access. Regular visits were made to each location once a week. The geographic and ecological characteristics of the sampling sites, including GPS coordinates, elevation, habitat type, and vegetation density, are provided in Table 2. The observation sites were selected opportunistically along the sampling routes, based on habitat representation and accessibility, rather than at fixed or predetermined distances. Representative photographs of the main habitat types surveyed during fieldwork are provided in Fig. 4. Observations were carried out on sunny days and hours (between 09:00 and 17:00). The visit of each flowering plant by pollinators was observed for 5 min (O’Connor et al., 2019), and the observed pollinator species and visit frequencies were recorded. Standard observation forms, developed based on the methodologies of the University of Illinois (2021) were used to record detailed information such as GPS coordinates of the location, date, time, weather conditions, plant species, and pollinator species. To better understand plant–pollinator interactions, all observations were documented with photographs. Plant samples necessary for identification were collected and prepared as herbarium material.

**Table 1:** June 2021 climate data-Türkmen mountain.

Parameter	Value	Unit	Description
Average air temperature	17.8	^∘^C	June 2021
Average relative humidity	71.9	%	June 2021
Total precipitation	55.8	mm	June 2021
Total sunshine duration	210.1	hours	June 2021
Soil temperature (20 cm)	20.3	^∘^C	June 2021
Average wind speed	3.34	m/s	Calculated average
Average solar radiation	6.02	kW/m^2^	Calculated average

**Table 2:** General geographic and ecological characteristics of the sampling sites in the Turkmen Mountain region.

Site Code	Approximate location	Elevation	Habitat type	Vegetation density	Location description
S1	39.724235, 30.416612	855 m	Roadside	Moderate	Sloping and rocky terrain with moderately dense herbaceous vegetation
S2	39.693042, 30.382841	853 m	Roadside	Moderate	Dry, gently sloping roadside with moderately dense herbaceous vegetation
S3	39.684864, 30.387802	879 m	Roadside	High	Gently sloping roadside with dense herbaceous vegetation and abundant flowering plants
S4	39.651626, 30.394762	829 m	Rural settlement edge	High	Located between a railway line and a rural road, characterized by dense herbaceous vegetation with flowering plants
S5	39.626762, 30.362661	858 m	Rocky area	Low	Sloping rocky surface with sparse vegetation
S6	39.602813, 30.364292	888 m	Roadside	High	Roadside area characterized by dense herbaceous vegetation with flowering plant species
S7	39.578704, 30.353748	970 m	Roadside	High	Roadside area characterized by high vegetation density and localized water accumulation
S8	39.546783, 30.498701	1,094 m	Agricultural area (roadside)	Moderate	Flowering plants occurring within and around a ploughed agricultural field with high species diversity
S9	39.519947, 30.478649	1,064 m	Grassland (sloped terrain)	High	Sloped area with dense herbaceous and flowering vegetation
S10	39.465621, 30.428489	1,337 m	Roadside	High	Gently sloping, edge of maquis habitat
S11	39.459194, 30.419691	1,411 m	Forest edge	High	Sloping, high-elevation forest edge
S12	39.425884, 30.413396	1426 m	Forest understory	High	High-elevation, shaded Scots pine understory
S13	39.377979, 30.601018	990 m	Disturbed roadside habitat	Low	Gently sloping, rocky disturbed roadside area

### Data analysis and evaluation

The data collected from field studies were first analyzed in terms of plant–pollinator relationships. Plants for which no pollinator visits were observed were excluded from the evaluation. Among the remaining species, those with potential for use in urban landscapes were selected based on their aesthetic properties (form, color, texture), ecological requirements (water, light, soil, *etc*.), phenological characteristics, and sustainability criteria. Plant identifications were made using Davis’s “Flora of Turkey and the East Aegean Islands” and current botanical literature. Pollinator species were identified with the help of relevant taxonomic keys and expert opinions. For each plant species, potential urban landscape uses (flower beds, rock gardens, borders, ground cover, *etc*.) were determined, and usage recommendations were developed.

All statistical analyses were performed using R version 4.2.1 (R Core Team, 2022). A *p* < 0.05 value was considered statistically significant for all tests. Descriptive statistics such as mean, standard deviation (SD), and coefficient of variation (CV = (SD/mean) × 100) were calculated to characterize the distribution of Pollinator Activity Index (PAI) values across the 16 plant families where pollinator activity was observed.

All correlation analyses were conducted at the plant family level (*n* = 16), based on families in which pollinator activity was recorded, rather than at the individual plant species level. Given the relatively small sample size (*n* = 16), correlation coefficients were interpreted cautiously, and only coefficients of approximately *r* ≥ 0.50 were considered indicative of statistically meaningful relationships at *p* < 0.05. Correlation analyses were performed to investigate the relationships between variables. Prior to the analysis, the normality assumption for all continuous variables was assessed using the Shapiro–Wilk test. Pearson’s moment correlation (r) was used for relationships where both variables showed a normal distribution. This included the analysis of pollinator activity against environmental variables (temperature, humidity), habitat characteristics (elevation, vegetation density), and the relationship between PAI and the number of plant species per family. For relationships involving at least one variable that did not follow a normal distribution, the nonparametric Spearman rank correlation (*ρ*) was used. Since pollinator diversity data did not follow a normal distribution, this method was preferred in the analysis of the relationship between PAI and pollinator diversity (number of visiting pollinator families).

Calculation of the Morphological Compatibility Index (MCI) A MCI was calculated to quantify the degree of physical adaptation between plants and the pollinators observed. For each unique plant–pollinator interaction observed, a binary compatibility score was assigned based on key morphological traits known to influence pollination success. Interactions were scored as ‘1’ (compatible) when the primary feeding organ of the pollinator (*e.g.*, proboscis length, body size) was compatible with the key dimensions of the flower (*e.g.*, corolla depth, diameter), enabling effective pollen transfer. When there was a clear morphological mismatch that could prevent successful pollination, the interaction was scored as ‘0’ (incompatible). The overall MCI for the ecosystem was calculated as the average of these binary scores across all unique plant–pollinator interactions observed, representing the proportion of morphologically well-matched interactions.

Calculation of the Phenological Overlap Ratio (POR) The temporal synchronization between plant flowering and pollinator activity was measured using a POR. For each plant species observed for pollinator visits, the total number of days recorded in flower during the June 2021 study period was defined as D_flower_. Within this flowering period, the number of days on which at least one known pollinator species was also actively observed in the study area was defined as Overlap. For each plant species, POR was calculated as the D_flower_/D_overlap_ ratio. The 82% POR reported in the results represents the average of the POR values of these individual species, weighted by the number of observation days, in order to account for differences in the number of observation days for each species.

### Pollinator activity index calculation

In this study, the Basic Pollinator Activity Index (PAI) was developed to quantitatively evaluate the pollinator attractiveness and activity of plant families. PAI integrates biological and environmental factors influencing plant–pollinator interactions and was constructed by adapting (Kevan & Baker, 1983; Potts et al., 2010). The parameters included in the index, their calculations, and their relative contributions to PAI are presented in Table 3.

**Table 3:** The parameters, its descriptions and their effect on PAI.

Parameter	Description	Formula/Value	Notes	Parameter	PAI effect	Status
PAI (Pollinator Activity Index)	Basic pollinator activity index	$\mathrm{PAI}= \frac{(\mathrm{FV}\times \mathrm{PE}\times \mathrm{PD})}{(\mathrm{L}\times \mathrm{CP}\times \mathrm{EFC})} $	An index that integrates biological and environmental factors.			
FV (Family Value)	Family diversity	$\mathrm{FV}= \frac{\text{The number of plant species in the family}}{\text{Total number of plant species observed}} $	In the range of 0–1; high values indicate family dominance	**FV ↑**	Increase	High family diversity
PE (Pollinator Existence)	Pollinator visit status	$PE= \left\{ \begin{array}{@{}l@{}} \displaystyle 1(\mathrm{Yes}) \\ \displaystyle 0(\mathrm{No}) \end{array} \right. $	Binary indicator	**PE= 1**	Increase	There is a visit by the pollinator
PD (Polinator Diversity)	Pollinator family diversity	$\mathrm{PD}= \frac{\text{Number of visiting pollinator families}}{\text{Total number of plant species observed}} $	In the range of 0–1; reflects high value diversity.	**PD ↑**	Increase	Various pollinator families
CP (Climatic Parameters Coefficients)	Average of climate parameters	$\mathrm{CP}= \left( \frac{\sum \mathrm{R}}{31} \right) \mathrm{X} \left( \frac{\sum \mathrm{P}}{30} \right) \mathrm{X}( \frac{\sum \mathrm{W}}{30} )$	R: Solar radiation (W/m^2^), P: Precipitation (mm), W W: Wind speed (m/s).	**CP ↑**	Decrease	Adverse climate conditions
EFC (Environmental Factor Coefficients)	Environmental parameters	$EFC= \frac{TxHxS}{1000} $	T: Temperature (°C), H: Humidity (%), S: sunshine duration (hour).	**EFC ↑**	Decrease	Extreme heat/humidity or low sunlight exposure

PAI Formula:

\begin{eqnarray*}PAI= \frac{FV\times PE\times PD}{L\times CP\times EFC} \end{eqnarray*}

Family Value (FV): The ratio of the number of plant species within a given family to the total number of plant species observed, reflecting the diversity and relative dominance of that family within the vegetation.

\begin{eqnarray*}FV= \frac{\text{The number of plant species in the family}}{\text{Total number of plant species observed}} \end{eqnarray*}

FV values range between 0 and 1, with higher values indicating families that are more widespread and structurally important within the ecosystem.

Pollinator Existence (PE): A binary indicator describing whether pollinators were observed visiting a given family during the survey period. If no pollinator visits were recorded, PAI was set to zero.

\begin{eqnarray*}PE= \left\{ \begin{array}{@{}l@{}} \displaystyle 1(\text{presence of pollinators}) \\ \displaystyle 0(\text{absence of pollinators})  \end{array} \right. \end{eqnarray*}

Polinator Diversity (PD): The ratio of the number of pollinator families visiting a given plant family to the total number of pollinator families recorded during the study, representing pollinator diversity associated with that family.

\begin{eqnarray*}PD= \frac{\text{Number of visiting pollinator families}}{\text{Total number of plant species observed}} \end{eqnarray*}

The PD value range between 0 and 1, with higher values indicating greater pollinator diversity.

It should be noted that PAI values were calculated at the family level. Consequently, a single pollinator individual could visit multiple species within the same family during the observation period, potentially inflating family-level PAI estimates, particularly for species-rich families such as Fabaceae. This potential pseudo-replication was acknowledged and considered when interpreting PD and overall PAI values.

Number of Locations (L): The total number of sampling locations included in the study (*n* = 13).

### Climate and environmental coefficients

Climatic Parameter Coefficient (CP): A composite coefficient incorporating monthly averages of solar radiation (R), precipitation (P), and wind speed (W), calculated using 30-31-day mean values. High precipitation and wind speeds can restrict pollinator activity, leading to increased CP values and, consequently, lower PAI scores.

\begin{eqnarray*}CP= \frac{\Sigma R}{31} \times \frac{\Sigma P}{30} \times \frac{\Sigma W}{30} \end{eqnarray*}

Environmental Factor Coefficient (EFC): A coefficient combining average temperature (T), relative humidity (H), and total sunshine duration (S). To standardize values, EFC was normalized by dividing by 1000.

\begin{eqnarray*}EFC= \frac{T\times H\times S}{1000} . \end{eqnarray*}

## Results

**Figure 5:**
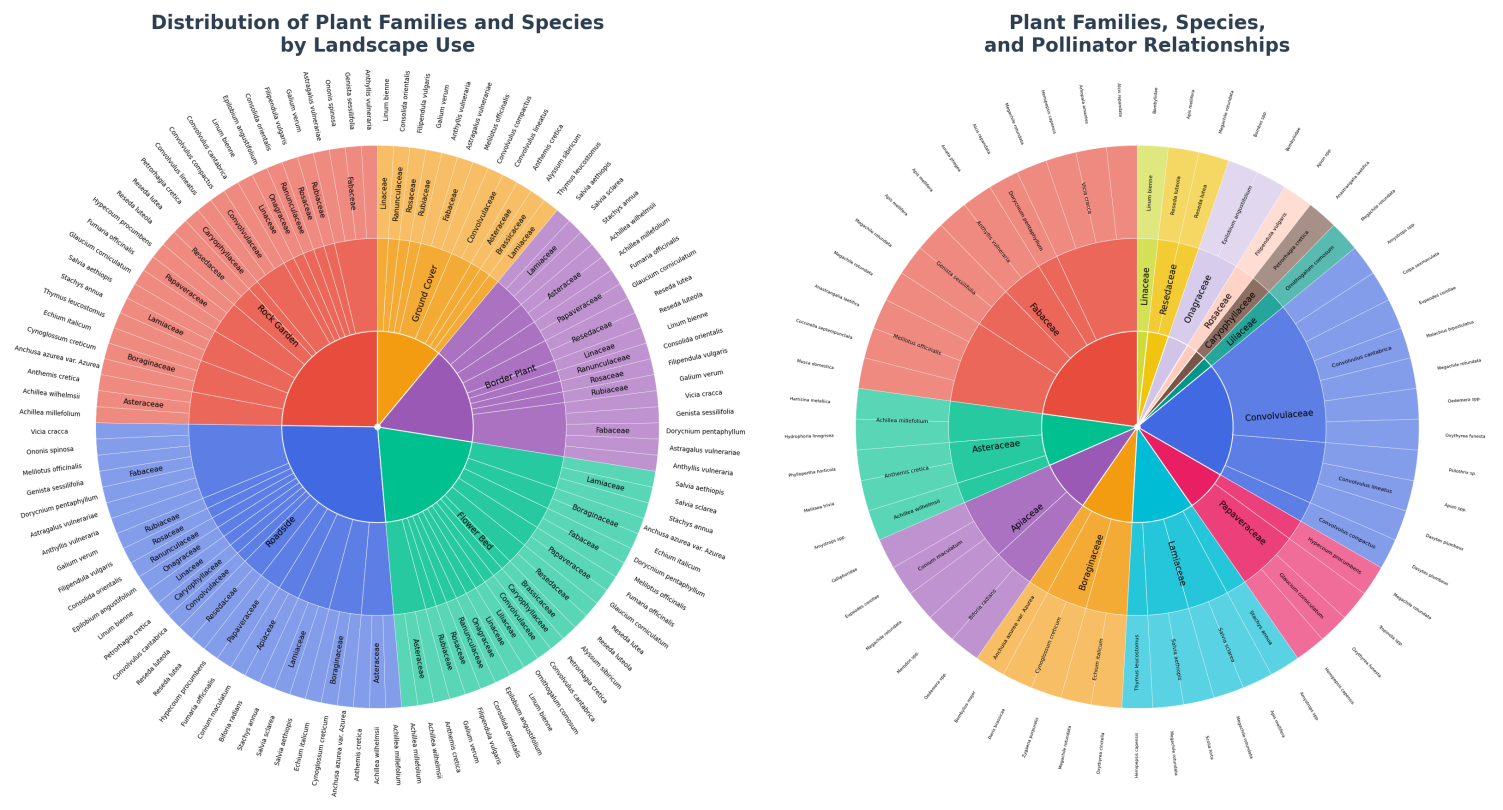
Plant–pollinator relationships and landscape use recommendations for plants.

As a result of field studies conducted in June 2021 in Eskisehir’s Turkmen Mountain, 57 flowering plant species were observed at 13 different locations, and pollinator activities were detected in 35 of them. These 35-plant species belonged to 16 different families, and a total of 30 genera of pollinators from 23 families were observed. The data obtained allowed for a detailed mathematical analysis of plant–pollinator relationships (Fig. 5).

**Figure 6:**
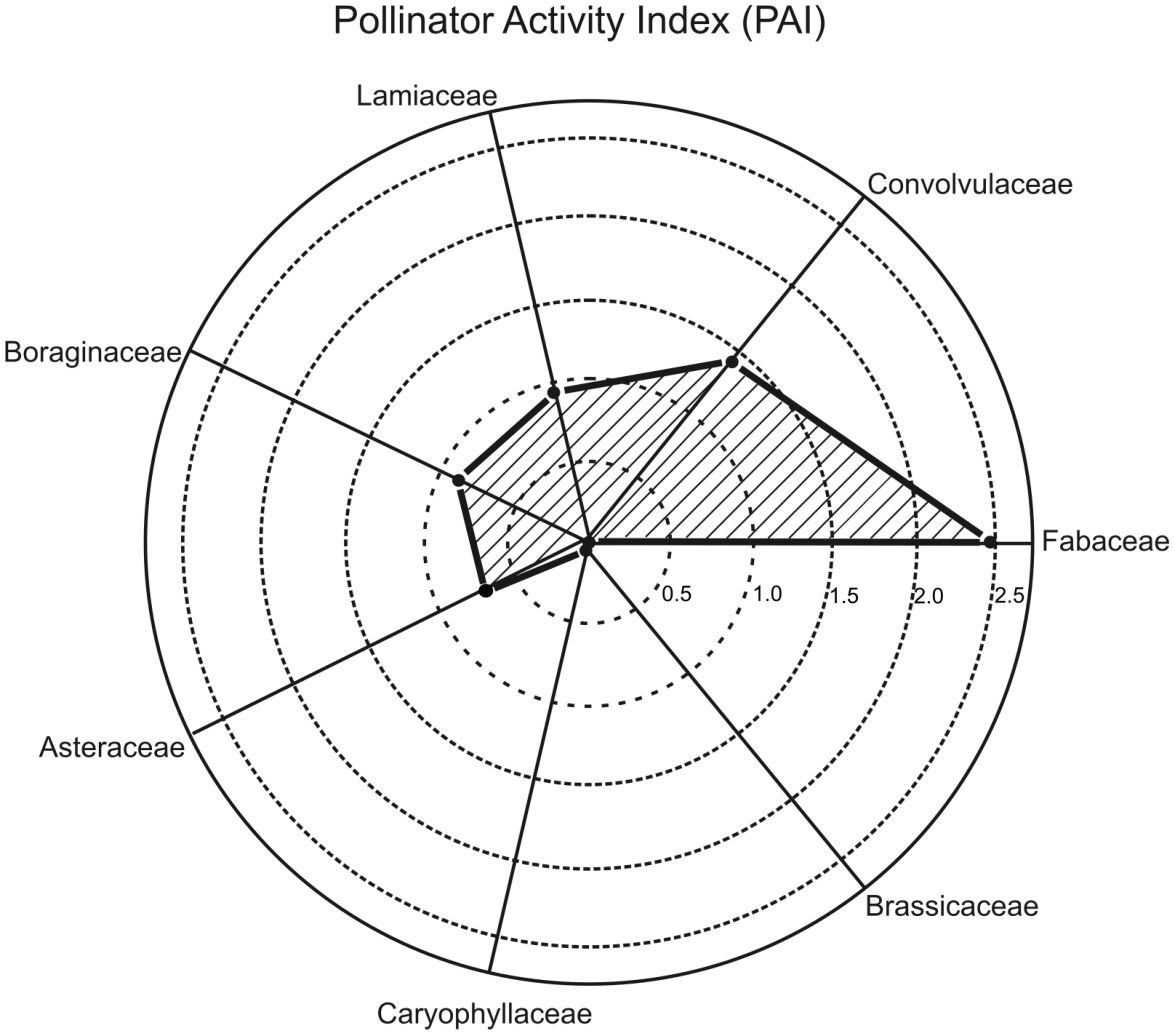
Pollinator activity index diagram by plant family.

Analyses performed using the PAI enabled the evaluation of pollinator activity at the family level (Table 4). The Fabaceae family was identified as having the highest pollinator activity with a PAI value of 2.484 × 10^−5^ (Fig. 6). This is directly related to the family attracting six different pollinator families with its seven-plant species (Table 4). In particular, the intensive visits of important pollinator species such as *Apis mellifera* and *Megachile rotundata* to Fabaceae species reveal that this family plays a critical ecological role (Fig. 5).

**Table 4:** PAI results by plant family.

Family	FV (family value)	PE (pollinator existence)	PD (pollinator diversity)	PAI result (×10^−5^)	Comment
Fabaceae	7/35	1	6/23	2.484	Highest PAI: Both plant species diversity (FD) and pollinator diversity (PC) are high.
Convolvulaceae	3/35	1	8/23	1.425	Attracts the highest pollinator diversity despite a low number of plant species.
Lamiaceae	4/35	1	4/23	0.945	Moderate level of FD and PC.
Boraginaceae	3/35	1	5/23	0.890	Moderate FD, relatively high PC.
Asteraceae	3/35	1	4/23	0.712	Combination of low FD and PC.
Caryophyllaceae	1/35	1	1/23	0.059	One of the lowest FD and PC values.
Brassicaceae	1/35	0	0/23	0.000	No pollinator visit detected during observation (PV = 0).

**Figure 7:**
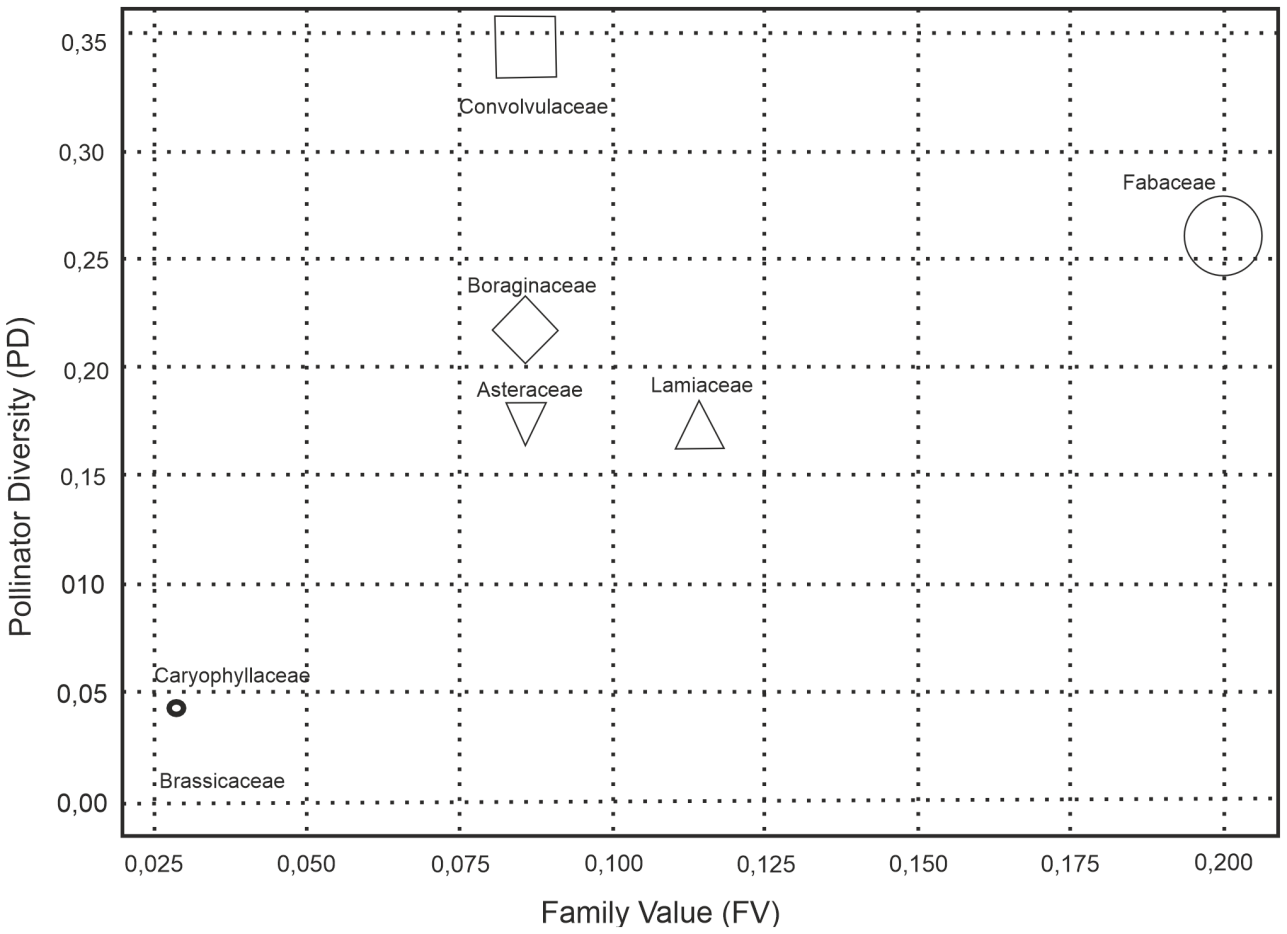
Pollinator diversity and family value relationship.

The Convolvulaceae family, with a calculated PAI value of 1.425 × 10^−5^, was identified as the family with the second-highest pollinator activity (Fig. 6). Despite containing only three plant species, its ability to attract eight different pollinator families (Table 4) shows that this family plays an important ecological role and highlights the decisive effect of flower morphology and nectar properties on pollinator diversity (Fig. 7).

The effects of meteorological parameters on pollinator activity were evaluated using correlation analyses at the plant family level (*n* = 16). A positive correlation was detected between air temperature and pollinator activity (*r* = 0.56, *p* < 0.05), with peak activity observed within the temperature range of 17–19 °C. In contrast, the correlation between relative humidity and pollinator activity was weaker (*r* = 0.48) and did not reach statistical significance at *p* < 0.05. Mean wind speed (3.34 m/s) and solar radiation (6.02 kW/m^2^) values were recorded during the observation period (Table 5).

**Table 5:** The effect of meteorological parameters on pollinator activity.

Factor	Correlation type	Value	Comment
Temperature	Pearson (r)	+0.56	Positive relationship; activity increases with temperature (optimal range 17–19 °C).
Humidity	Pearson (r)	+0.48	Moderate positive relationship; highest activity observed at 71.9% humidity.
Vegetation density	Pearson (r)	+0.62	Strong positive relationship; dense vegetation increases pollinator activity.
Altitude gradient	Pearson (r)	−0.45	Negative relationship; activity tends to decrease as altitude increases.

Statistical analyses showed that PAI values among families were distributed over a wide range (mean: 0.931 × 10^−5^, standard deviation: 0.876 × 10^−5^). A high coefficient of variation (94.1%) reflects the significant differences in the pollinator attraction potentials of the families. The strong positive correlation (*r* = 0.82, *R*^2^ = 0.672, *p* < 0.01) between the number of species and PAI values mathematically confirms the significant impact of plant diversity on pollinator activity. The high Spearman correlation (*ρ* = 0.79, *p* < 0.01) between pollinator diversity and PAI reveals the complex structure of ecological interactions (Table 6).

**Table 6:** Comprehensive data compilation.

Data category	Data point	Value	Unit/Format
General ecology	Dependence of flowering plants on insects	∼75	%
General ecology	Dependence of food production on pollination	35	%
General ecology	Honeybee colony decline (Central Europe, 1947-2005)	25	%
General ecology	Honeybee colony decline (USA, 1947-2005)	59	%
Study parameter	Number of observation locations	13	Count
Study parameter	Total plant species observed	57	Count
Study parameter	Plant species with pollinator activity	35	Count
Study parameter	Number of plant families studied	16	Count
Study parameter	Number of pollinator families observed	23	Count
Climate	Average air temperature (June 2021)	17.8	^∘^C
Climate	Average relative humidity (June 2021)	71.9	%
Climate	Total precipitation (June 2021)	55.8	mm
PAI result	Fabaceae	2.484 × 10^−5^	PAI
PAI result	Convolvulaceae	1.425 × 10^−5^	PAI
PAI result	Lamiaceae	0.945 × 10^−5^	PAI
PAI result	Boraginaceae	0.890 × 10^−5^	PAI
PAI result	Asteraceae	0.712 × 10^−5^	PAI
PAI result	Caryophyllaceae	0.059 × 10^−5^	PAI
PAI result	Brassicaceae	0.000 × 10^−5^	PAI
Statistical analysis	Temperature *vs.* activity correlation	0.56	r
Statistical analysis	Humidity *vs.* activity correlation	0.48	r
Statistical analysis	Altitude *vs.* activity correlation	−0.45	r
Statistical analysis	Vegetation density *vs.* activity correlation	0.62	r
Statistical analysis	Plant species count *vs.* PAI correlation	0.82	r
Statistical analysis	Plant species count *vs.* PAI R-Square	0.672	R^2^
Statistical analysis	Pollinator diversity *vs.* PAI correlation	0.79	*ρ*
Ecological index	Morphological adaptation index (Avg.)	0.73	Index value
Ecological index	Phenological overlap ratio	82	%
Landscape recommendation	Recommended family combination (Fabaceae)	35	%
Landscape recommendation	Recommended family combination (Convolvulaceae)	25	%
Landscape recommendation	Recommended family combination (Lamiaceae)	20	%

The morphological and phenological dimensions of plant–pollinator compatibility were also analyzed. The average morphological compatibility index was calculated as 0.73, indicating a high level of adaptation of the examined plant species to pollinators. Additionally, an 82% phenological overlap rate indicates strong synchronization between plant flowering periods and pollinator activity periods (Table 6).

When examining the effect of habitat characteristics on pollinator activity, a negative correlation was observed between pollinator activity and elevation (*r* = −0.45), indicating a decrease in pollinator activity with increasing altitude. In contrast, a positive correlation was detected between pollinator activity and vegetation density (*r* = 0.62), with higher activity recorded in areas with denser vegetation (Table 5).

**Figure 8:**
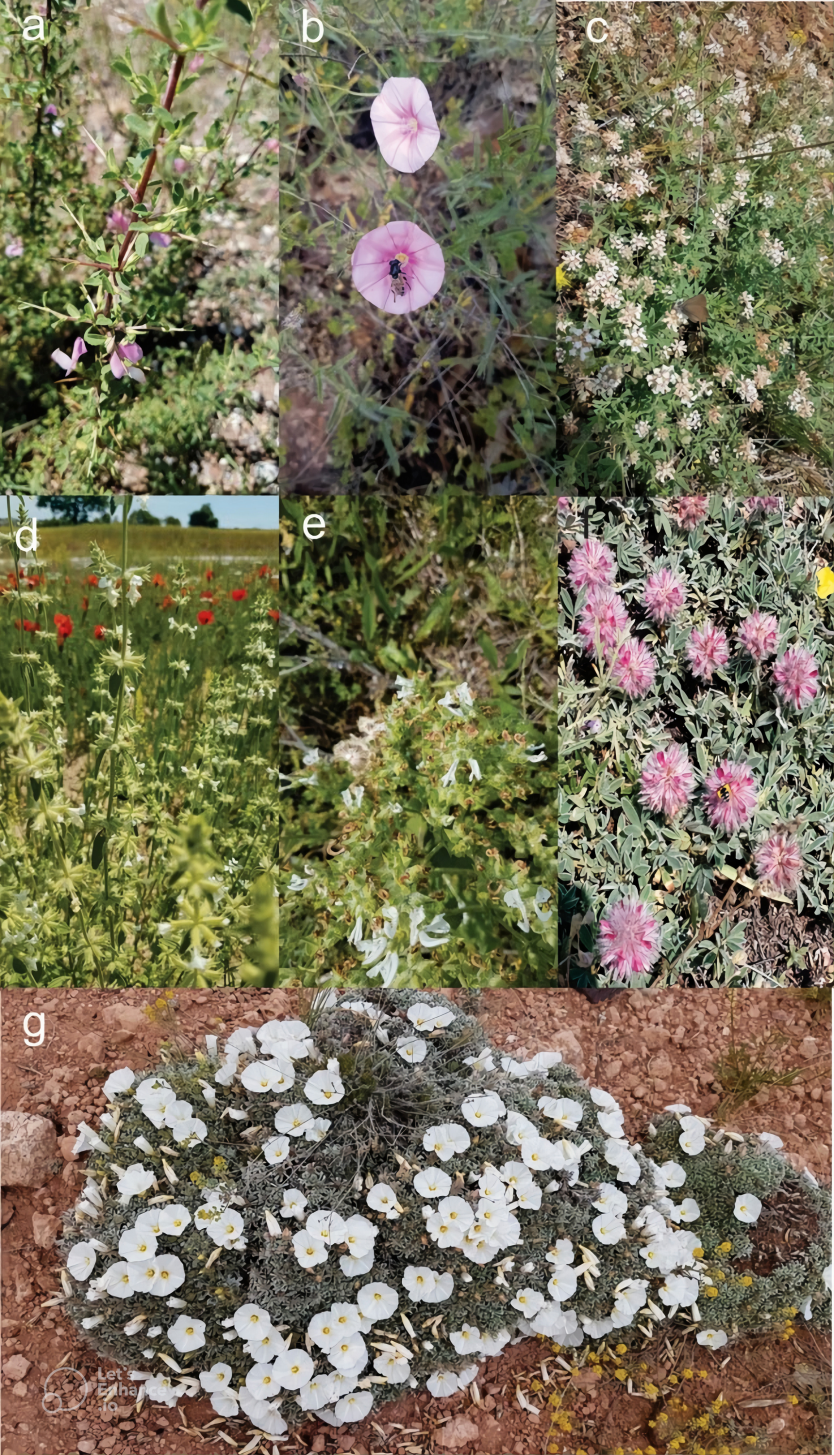
(A) *Ononis spinosa*, (B) *Convolvulus cantabrica*, (C) *Dorycnium pentaphyllum*, (D) *Stachys annua*, (E) *Salvia aethiopis*, (F) *Anthyllis vulneraria*, (G) *Convolvulus compactus*) (Orij, 2021).

Figure 8 presents representative examples of native plant species observed in the study area that illustrate different landscape functions, and is not restricted to the subset of species selected for management recommendations in Table 7. The potential for using the plant species observed in Turkmen Mountain in urban landscapes was also evaluated (Fig. 5). For example; *Ononis spinosa* can be used in rock gardens, medians, and roadside areas due to its upright structure (Fig. 8A). With its horizontal stem, upright flower stalks, and striking pink flowers, *Convolvulus cantabrica* is suitable for rock gardens, flower beds, medians, and roadside borders (Fig. 8B). *Dorycnium pentaphyllum*, as a border plant, it can be used in low-maintenance areas and along roadsides (Fig. 8C) *Stachys annua*, with its upright, multi-branched structure, can be used alone or in groups in flower beds, in rock gardens, as a border plant, and also on roads and medians (Fig. 8D). *Salvia aethiopis* can be used in flower beds, rock gardens, as a border plant, in medians, and along roadsides. In addition to being heat-tolerant, *S. aethiopis* can also withstand frost. Its sharp-lobed, rosette-shaped gray leaves make it suitable for use as a border plant, and it can also create contrast with other green or dark-colored plants (Fig. 8E). *Anthyllis vulneraria* is a plant that can be used in rock gardens, medians, and roadsides. *A. vulneraria,* which can grow in poor and dry soils, can be used to create rock gardens and meadows (Fig. 8F). *Convolvulus compactus* with its gray-green leaf color, compact structure, dwarf form, and beautiful flowers, it can be used in rock gardens and as ground cover (Fig. 8G).

**Table 7:** Selected native plant species with high pollinator support potential and recommended landscape functions for urban green spaces.

Family	Plant species	Main pollinator groups	Urban application/ landscape function
Asteraceae	*Achillea millefolium*	Beetles, flies	Urban meadows and low-maintenance planting
	*Anthemis cretica*	Lepidoptera, Flies, Beetles	Urban meadows and low-maintenance planting
Boraginaceae	*Anchusa azurea*	Flies, Lepidoptera	Accent and habitat-supporting planting
Convolvulaceae	*Convolvulus cantabrica*	Bees, Beetles, Flies, Wasps	Accent and habitat-supporting planting
Fabaceae	*Dorycnium pentaphyllum*	Bees, Lepidoptera	Habitat-supporting and structural planting
	*Vicia cracca*	Bees, Wasps, Lepidoptera	Habitat-supporting and connective planting
Lamiaceae	*Thymus leucostomus*	Bees, Wasps	Xeric and low-maintenance planting
Papaveraceae	*Glaucium corniculatum*	Beetles, Wasps	Accent and habitat-supporting planting (restricted use)

## Discussion

Pollinators play a critical role in the sustainability of plant resources and the conservation of biodiversity in urban ecosystems. Furthermore, urban green spaces are defined as important areas for pollinator conservation. Silva et al. (2023) state that urban green spaces function as “hotspots” for pollinators and that these areas offer both floral resources and nesting sites for them. Additionally, it is emphasized that to ensure the continuity of resources provided by ornamental plants in these areas, species that flower throughout the year should be integrated (Llodra-Llabrés & Carinanos, 2022). On the other hand, it is stated that urban landscapes with natural green spaces support higher pollinator diversity and are critically important for the sustainability of pollinator populations (Ulrich & Sargent, 2025). All these views emphasize the critical role of using pollinator-supporting plant species in urban landscape designs and the role of urban green spaces in supporting pollinator communities by providing the necessary resources for their survival and reproduction.

In our study, the interactions between flowering plants in the Turkmen Mountain flora and pollinators, as well as the effects of climatic factors on these interactions, were examined, and plants with potential for landscape use were identified.

According to our findings, plants belonging to the Fabaceae and Convolvulaceae families, respectively, were found to have the highest pollinator activity. The preference of pollinators for flowering plants in the Fabaceae family due to their high-protein pollen is supported by various scientific studies (Tuylu & Sorkun (2006), Cinbirtoglu & Guney (2021), Khalil, Sykes & Russo (2023). The fact that Fabaceae plants showed the highest pollinator activity in our study supports this view.

Convolvulaceae is a large plant family with 60 genera and approximately 2,000 species worldwide (Simões et al., 2022). In Turkey, this family includes 6 genera and 61 species (Guner et al., 2012). Species of this family show significant diversity in terms of flower morphology, reproductive systems, and pollinator relationships (Hassa, Traiperm & Stewart, 2023). For example, some species have a high degree of pollinator dependency, while others can self-pollinate. These differences vary depending on flower morphology and reproductive strategies (Demirpolat & Kilic, 2021; Hassa, Traiperm & Stewart, 2023). Although scientific studies on Convolvulaceae family and pollinator interactions are limited, Olesen et al. (2007) state that the bell-funnel shaped flower morphology in this family allows access to most of the animals visiting the flowers and that these flowers have a specialized visitor fauna consisting of long-tongued animals. In a study conducted by Hassa, Traiperm & Stewart (2023) in Northeast Thailand, 15 different Convolvulaceae species were examined, and it was observed that bee species such as Lasioglossum, Amegilla, Apis, and Meliponine bees were the main pollinators, while one species was visited by twilight-flying butterflies and nocturnal moths. In our study, although the number of plants belonging to the Convolvulaceae family was limited, it had a very rich structure in terms of pollinator diversity. *Convolvulus cantabrica* stands out as the species that attracts the most pollinators. Its pollinators include *Amystrops spp., Colpa sexmaculata, E. corollae, Malachius bipustulatus, M. rotundata, Oedemera spp., Oxythyrea funesta*, and *Psilothrix sp*. On the other hand, the pollinator of *Convolvulus compactus* is *Dasytes plumbeus*, while *Convolvulus lineatus* is visited by *Apion spp.* and *Dasytes plumbeus*. The interaction of *Convolvulus cantabrica* with a wide range of pollinators, in particular, indicates that this species plays an ecologically important role and constitutes a significant resource for various pollinator species. The differences among the pollinators of other species, however, highlight that each species can adapt specifically to different pollinator groups, thus emphasizing the effect of factors like flower morphology, color, and nectar production on pollinator preferences.

In terms of pollinator activity, following the Fabaceae and Convolvulaceae families, the Lamiaceae (moderate pollinator diversity), Boraginaceae, and Asteraceae (low pollinator diversity) families were also found to contribute to pollinator-plant interactions. This may be due to the varying pollinator attraction capacities of plants belonging to these families, depending on their flower morphology, nectar production, and other attractive features. Khalil, Sykes & Russo (2023) state that flowers of the Lamiaceae family are preferred by pollinators due to their significant nectar production, and flowers of the Asteraceae family are preferred for their attractive, open inflorescence and long flowering periods. In our study, pollinator species observed on plants of the Lamiaceae family include *Megachile rotundata*, *Apis mellifera*, *Hemipepsis capensis*, and *Scolia hirta*. *A. mellifera* is a common pollinator in Lamiaceae species due to its wide distribution and generalist feeding behavior (Venkatesh et al. (2022), Campolo et al. (2015).

*M. rotundata* is a leaf-cutter bee species known for its nectar and pollen collection (Cane, Gardner & Harrison, 2011), contributing to the pollination of Lamiaceae flowers. Wasps such as *Hemipepsis capensis* and *Scolia hirta* can increase the reproductive success of these plants by mediating pollen transfer during flower visits. These findings show that Lamiaceae species benefit from different pollinator groups in their pollination process and that these interactions are important for plant reproduction. Similarly, Venkatesh et al. (2022) state that Lamiaceae species are highly dependent on bee pollinators for their reproduction and regeneration processes.

Some bee species have been reported to maintain a sustainable presence in urban areas (Marquardt et al., 2021; Silva et al., 2023). However, it is emphasized that other pollinator groups such as solitary bees, Diptera, Lepidoptera, and Coleoptera are more sensitive to urbanization (Marquardt et al., 2021). Therefore, studies on pollinator-plant interactions in urban areas should be planned with strategies aimed at protecting these sensitive groups. This approach will contribute to increasing pollinator diversity.

There is a significant interaction between pollinators and climate. Climate changes, especially temperature increases and changes in precipitation patterns, can directly affect the distribution and activity of pollinators. For example, Nehybová & Horák (2024) reveal in their research that instantaneous weather factors such as temperature and wind have a direct and strong effect on pollinators. They stated that temperature increases and wind speed changes significantly affect pollinator behavior and movements. On the other hand, our study found a positive relationship between temperature and pollinator activity. That is, as temperature increases, pollinator activity also increases. Specifically, peak pollinator activity was observed within the temperature range of 17–19 °C, indicating that temperature plays a critical role in regulating pollinator efficiency. In our study, the effect of the humidity factor on pollinator activity was found to be moderate. However, the relationship between relative humidity and pollinator activity was weaker and did not reach statistical significance when evaluated at the family level. A study by Bailey, Lerer & Mills (1982) stated that pollination activity showed a strong relationship with relative humidity, but this relationship misrepresented the humidity response. When temperature effects were considered, the direct influence of atmospheric humidity on pollinator activity appeared to be limited, and this finding reveals that caution should be exercised when evaluating the effects of atmospheric humidity on biological activity. Wind speed and solar radiation are also factors that should not be ignored. Furthermore, climatic stress factors such as extreme weather events and drought can threaten pollinator populations by making it difficult for them to access food sources. This can negatively affect the pollination success of plants and consequently the continuity of ecosystem services. Therefore, in ecological improvement and pollinator conservation studies, environmental and climatic variables should be evaluated together and as accurately as possible, with particular attention to site-specific climatic conditions.

Ectothermic (body temperature dependent on the environment) insect pollinators are highly sensitive to environmental (abiotic) conditions. However, the ability of pollinators to be active depends not only on environmental conditions but also on biological (biotic) factors such as the presence of host plants. High-altitude regions are places where sharp changes in environmental conditions (environmental gradients) are observed over short distances. Thanks to these features, they offer an ideal natural laboratory for examining the relative effects of abiotic (*e.g.*, temperature, humidity) and biotic (*e.g.*, interactions with plants) factors on pollinators (Coates et al., 2024). In this context, the fact that our study area represents a semi-natural, high-altitude ecosystem provides a valuable framework for disentangling the effects of climatic variables and plant–pollinator interactions under relatively controlled conditions. Similarly, the fact that our study area is a high-altitude region compared to the urban environment shows that it offers an ideal setting for examining the effects of such environmental gradients and supports the correctness of our site selection.

At the same time, these characteristics underline the importance of cautious interpretation when extrapolating the findings to urban ecosystems, where environmental gradients and stressors differ substantially. Therefore, the observed correlations between pollinator activity and environmental variables should be interpreted with caution, as unmeasured confounding factors such as floral resource distribution, microhabitat heterogeneity, and short-term weather variability may also influence pollinator behavior.

Pollinator abundance can vary depending on altitude. Generally, as altitude increases, the abundance of both flowers and pollinators decreases, because high altitudes have more challenging environmental conditions for plants and pollinators (Ren et al., 2018; Tong, Wu & Huang, 2021). Medan et al. (2002) examined angiosperm flowers and pollinator communities at different altitudes in the Mendoza Andes and recorded more plant and pollinator species and more intense interactions at lower altitudes. Similarly, Zhao et al. (2016), in their study conducted at altitudes of 2,725–3,910 m, concluded that pollinator diversity was higher at lower altitudes. In our study, pollinator abundance showed a decreasing trend with increasing altitude, and a moderate negative correlation (*r* = −0.45) was observed along the elevation gradient. Although this relationship did not reach statistical significance at *p* < 0.05, it is consistent with patterns reported in previous studies.

Plants are dependent on pollinators for their reproductive processes. Pollinators not only ensure gamete transfer by carrying pollen but also shape this interaction through their behaviors and physical structures by interacting with the characteristics of flowers. The adaptation of plants to different pollinator species is considered one of the fundamental mechanisms that promote the diversification of angiosperms (flowering plants) and the formation of new species (speciation) (Schiestl & Schlüter, 2009; Sun, Gross & Schiestl, 2014). In our study, analyses indicated that plant diversity was strongly associated with pollinator activity, and the average morphological compatibility index was observed to be 0.73. This indicates a high level of adaptation of plants to pollinators. Additionally, an 82% phenological overlap rate reveals strong temporal compatibility between flowering periods and pollinator activity.

It has been stated that dense flower clusters attract more pollinators (Elliott & Irwin, 2009; Schmitt, 1983). Klinkhamer & De Jong (1990) state that in dense flower clusters, the proximity of plants to each other allows them to attract more pollinators than each plant could independently, while also emphasizing that these dense clusters can trigger competition among plants. That is, when a pollinator reaches a plant, it may tend to visit only a limited number of flowers instead of all the flowers on that plant. This can lead to a decrease in the probability of each flower being visited, despite an increase in the number of flowers. Consistent with these observations, our results indicate that pollinator activity tended to be higher in areas with dense vegetation.

This study was conducted in a semi-natural, high-altitude mountain ecosystem. Some of the plant species observed within the scope of the study are proposed as potential candidates for landscape applications in urban ecosystems, which differ markedly in terms of air quality, climatic parameters, and anthropogenic pressures. However, it is evident that such environmental differences between ecosystems may substantially influence plant–pollinator interactions. Therefore, although the proposed plant species and families exhibit high potential for supporting pollinators, their transfer to urban landscape contexts should be approached with caution, and additional experimental and observational studies conducted in urban environments are necessary to evaluate their adaptability and practical applicability under urban landscape conditions.

Based on pollinator activity, species richness, and observed plant–pollinator interactions, a selected list of native plant species with high potential for supporting pollinators and urban landscape use is presented in Table 7. Species included in this table were selected based on their interaction with three or more pollinator taxa, reflecting their relatively broad pollinator support capacity. This table provides a management-oriented summary by linking each species to its main pollinator groups and general landscape functions, such as habitat-supporting, low-maintenance, or accent planting. The proposed species are intended to complement urban green space designs by enhancing ecological functionality while acknowledging the need for site-specific evaluation and appropriate management under urban conditions. Despite exhibiting high pollinator visitation rates, *Conium maculatum* was not included in the list due to its toxic properties and limited suitability for urban landscape applications.

The results offer important implications for pollinator conservation in urban landscape design. Prioritizing plant families with high PAI values is a critical strategy for supporting sustainable pollinator populations. In particular, a balanced combination of families such as Fabaceae (35%), Convolvulaceae (25%), and Lamiaceae (20%) is important for increasing pollinator diversity and sustaining long-term pollination processes. These families support the continuity of ecosystem services by providing food resources and nesting sites for pollinators. However, since PAI was calculated at the family level, these results should be interpreted with caution, particularly for species-rich families, due to the potential pseudo-replication arising from multiple visits by the same pollinator individual. Accordingly, PAI should be regarded as a complementary indicator that captures general interaction patterns rather than a standalone metric representing all dimensions of plant–pollinator relationships. The application of the PAI in this study offers a methodological advantage over traditional monitoring approaches that rely solely on visitation frequencies. Previous studies have often evaluated pollinator preference and environmental variables as separate, independent datasets (Nehybová & Horák, 2024; Coates et al., 2024). However, the PAI integrates these dimensions into a single composite metric, weighting biological interaction (diversity and presence) against environmental constraints (temperature, wind, and humidity). This holistic approach is particularly applicable to urban landscape planning, where microclimatic heterogeneity (*e.g.*, urban heat islands, wind tunnels) significantly alters pollinator behavior compared to natural settings (Hennig & Ghazoul, 2011). By treating environmental suitability as a multiplicative coefficient, the PAI helps identify plant families that not only attract pollinators but do so effectively under prevailing climatic conditions, providing a more robust performance metric for ecological restoration and design (Dixon, 2009).

Nevertheless, the application of PAI in this study is subject to specific limitations that must be considered in future research. Firstly, calculating the index at the family level employs a ’higher-taxon surrogacy’ approach. While this provides practical, broad guidelines for planting design, it inevitably masks species-level specializations. Nevertheless, higher-taxon surrogacy has been shown to be a cost-efficient and reliable method for biodiversity monitoring, particularly in Mediterranean ecosystems (Mandelik, Roll & Fleischer, 2010). Future studies could refine the PAI by applying it at the genus or species level to increase taxonomic resolution. Secondly, the current calculation relies on data from the peak flowering season (June). As urban pollinator communities exhibit distinct seasonal shifts, relying on a single temporal snapshot may overlook plant species critical for early spring or late autumn resources (Plant et al., 2025). Therefore, we recommend that future applications of PAI utilize multi-season datasets to capture the full phenological range of urban ecosystems.

On the other hand, in plant selection, it is important to consider not only morphological characteristics but also ecological factors. Factors such as temperature tolerance, humidity adaptation, and wind resistance are important parameters that may influence both the ability of plants to adapt to local climatic conditions and pollinator activity. When evaluated together with site-specific conditions, such ecological characteristics play a fundamental role in creating a more sustainable urban landscape.

It is important to note that field data in this study were collected only in June 2021, corresponding to the peak flowering period. Therefore, conclusions regarding year-round flowering continuity and planting patterns are inferred from existing literature on plant phenology rather than directly derived from multi-season field observations. This constitutes a limitation of the study and highlights the need for future multi-season or multi-year research to validate these inferences.

Based on our findings, in plantings to be made in urban landscape designs, the use of plant species from at least three different families and ensuring phenological continuity may help pollinators to access food and pollen resources throughout the year. Phenological continuity can be achieved by overlapping the flowering periods of plants, which is expected to support the continuity of food availability for pollinators. These strategies have the potential to strengthen the adaptation ability of pollinator species and support ecosystem stability by providing habitat diversity. Overall, such approaches may provide an effective roadmap for increasing biodiversity and improving ecosystem services in urban areas.

## Conclusions

This study focused on the interactions between flowering plants that grow naturally in the Turkmen Mountains and the pollinators that visit these plants. In addition to highlighting the ecological importance of these plants, the study also draws attention to the potential importance of their use in urban areas, which are among the environments most affected by climate change, due to their low maintenance requirements and tolerance to extreme conditions. Based on observations conducted in June 2021, the findings suggest that plant families such as Fabaceae, Convolvulaceae, and Lamiaceae appear to exhibit relatively high pollinator activity. Additionally, the results suggest that climatic factors such as temperature, humidity, and light may influence pollinator behavior, and that a notable morphological and phenological compatibility between plants and pollinators was observed. In conclusion, the conservation of native plant species and their potential preference in urban green spaces may play an important role in supporting the sustainability of pollinator populations and the preservation of biodiversity.

## Supplemental Information

10.7717/peerj.21143/supp-1 Supplemental Information 1 Raw data

## Additional Information and Declarations

### Data Availability

The following information was supplied regarding data availability: The raw data is available in the Supplemental File.
